# The serum cholesterol level of choledocholithiasis patients was significantly lower than that of healthy people

**DOI:** 10.3389/fendo.2025.1552389

**Published:** 2025-05-20

**Authors:** Linzhen Li, Tulan Hu, Di Wang

**Affiliations:** ^1^ Departments of Gastroenterology, First Affiliated Hospital of Wannan Medical College, Wuhu, Anhui, China; ^2^ Departments of Gastroenterology, The Affiliated Hospital of Xuzhou Medical University, Xuzhou, Jiangsu, China

**Keywords:** choledocholithiasis, triglyceride, total cholesterol, high density lipoprotein, low density lipoprotein

## Abstract

**Background and purpose:**

The specific pathogenesis of choledocholithiasis is still unclear. The objective of this study was to investigate whether serum cholesterol level is related to the incidence of choledocholithiasis.

**Patients and methods:**

A total of 169 choledocholithiasis patients were include in this study. We investigated whether total cholesterol (TC), triglyceride (TG), high density lipoprotein (HDL-C), low density lipoprotein (LDL-C), TC/TG, LDL-C/HDL-C in choledocholithiasis patients differed from that in healthy people.

**Results:**

There were 64 male and 105 female patients. There were significant differences in TC (*P*<0.001), TG (*P*=0.006), HDL-C (*P*<0.001) and LDL-C (*P*=0.001) between the two groups. The TC, TG, HDL-C, LDL-C in the choledocholithiasis patients group were significantly lower than that in the healthy population. In the subgroup analysis, we further investigate whether above parameters in choledocholithiasis patients differed from that in healthy people by gender. There was no significant difference in TG (*P*=0.182), TC/TG (*P*=0.982), LDL-C/HDL-C (*P*=0.392) between the male choledocholithiasis patients group and the male healthy population group. There were significant differences in TC (*P*=0.001), HDL-C (*P*=0.014) and LDL-C (*P*=0.026) between the two groups. There were no significant difference in TC/TG (*P*=0.590), LDL-C/HDL-C (*P*=0.116) between the female choledocholithiasis patients group and the female healthy population group. There were significant differences in TC (*P*<0.001), TG (*P*=0.016), HDL-C (*P*<0.001) and LDL-C (*P*=0.009) between the two groups.

**Conclusions:**

The serum TC, HDL-C and LDL-C in choledocholithiasis patients were significantly lower than those in healthy people.

## 1 Introduction

Choledocholithiasis is a common digestive tract disease, which can be divided into primary and secondary choledocholithiasis according to the source. Primary stones usually form in cases of bile duct stasis or physiologic bile duct dilatation, both of which are more likely to form intrabile stones (1). Secondary choledocholithiasis is more common and is usually caused by a cholesterol-forming gallstone falling into the common bile duct (1). The prevalence of choledocholithiasis varies from country to country, ranging from 8% to 20% in symptomatic gallstones (2–6). A light and regular diet can help prevent common bile duct stones. Serious complications of choledocholithiasis include biliary pancreatitis and acute cholangitis, which can increase the mortality of patients (7). Choledocholithiasis is more common in older adults with choledochal physiologically dilated. Previous studies (8) have shown that in patients with prior cholecystectomy, common bile duct may physiologically expand to 10mm, leading to cholestasis and primary stone formation. Typical clinical symptoms of choledocholithiasis are pain in the upper abdomen, which may be accompanied by nausea and vomiting. The pain cannot be relieved by changing position and is not related to eating. Clinically, imaging tests that can be used to diagnose choledocholithiasis include transabdominal ultrasound, computerized tomography, magnetic resonance cholangiopancreatography, endoscopic retrograde cholangiopancreatography, percutaneous transhepatic cholangiography, laparoscopic ultrasound and endoscopic ultrasound (9–13).

Lipids are a general term for neutral fats (triglycerides and cholesterol) and lipids (phospholipids, glycolipids, sterols, steroids) in plasma, which are widely found in the human body. They are essential for the basic metabolism of living cells. In general, the main components of blood lipids are triglycerides and cholesterol, in which triglycerides are involved in energy metabolism in the human body, and cholesterol is mainly used for the synthesis of cell serous membranes, steroid hormones and bile acids. Cholesterol is involved in the development of atherosclerosis and is associated with acute ischemic arterial embolism events (14, 15). Since bile contains cholesterol components, whether abnormal cholesterol metabolism is involved in the formation of bile duct stones? The objective of this study was to investigate whether there were significant differences in serum cholesterol and triglycerides between choledocholithiasis patients and healthy people.

## 2 Materials and methods

### 2.1 Study design

The objective of this retrospective study was to investigate whether serum cholesterol level is related to the incidence of choledocholithiasis. The inclusion criteria were as follows: (1) patients who meet the diagnostic criteria for choledocholithiasis (magnetic resonance imaging or abdominal color Doppler ultrasound suggested choledocholithiasis, and ERCP confirmed), (2) blood was drawn for total cholesterol (TC), triglyceride (TG), high density lipoprotein (HDL-C), low density lipoprotein (LDL-C). The exclusion criteria were as follows: (1) have been or are taking lipid-lowering medications, (2) complicated with hematological diseases, coronary heart disease, cerebral infarction and other cardiovascular and cerebrovascular diseases, (3) previous cholecystectomy patients.

### 2.2 Patients and methods

A total of 169 choledocholithiasis patients (January 2022-July 2024) in the Department of Gastroenterology of the First Affiliated Hospital of Wannan Medical College were include in this study. Gender, age, TC, TG, HDL-C, LDL-C, TC/TG, LDL-C/HDL-C were recorded. To investigate differences in total cholesterol, triglyceride, high density lipoprotein, low density lipoprotein between choledocholithiasis patients and normal people, 169 patients who underwent physical examination in our hospital from January 2022-July 2024 were randomly selected and their age, sex and TC, TG, HDL-C, LDL-C, TC/TG, LDL-C/HDL-C were also recorded.

First, we investigated whether TC, TG, HDL-C, LDL-C, TC/TG, LDL-C/HDL-C in choledocholithiasis patients differed from that in healthy people. If there is a significant difference, in the subgroup analysis, we further investigate whether TC, TG, HDL-C, LDL-C, TC/TG, LDL-C/HDL-C in choledocholithiasis patients differed from that in healthy people by gender.

### 2.3 Ethical considerations

The research was performed according to the Declaration of Helsinki including patients’ consent. The study was approved by the local Ethics Committee.

### 2.4 Statistical analysis

Descriptive data are expressed in terms of ¯x ± s. All numerical variables were tested for normal distribution (Kolmogorov-Smirnov test). Independent-samples T test was used for parametric tests, and Chi-square test or Fisher’s exact test was used for categorical variables. SPSS 21.0 software was used for statistical analysis. A *P*-value < 0.05 indicated statistical significance.

## 3 Results

### 3.1 Clinical features of the 169 choledocholithiasis patients

A total of 169 choledocholithiasis patient were include in this study. There were 64 (37.87%) males and 105 (62.13%) females. The mean age was 56.26 ± 10.80 years old. The mean of TC, TG, HDL-C, LDL-C were 4.30 ± 1.03 mmol/L, 1.33 ± 0.76 mmol/L, 1.27 ± 0.37 mmol/L and 2.51 ± 0.77 mmol/L respectively. (**Table 1**).

**Table 1:** Clinical features of choledocholithiasis patients.

Sex (M/F)	64/105
Age (years): ¯x±s	56.26±10.80
TC:¯x±s , mmol/L	4.30±1.03
TG: ¯x±s , mmol/L	1.33±0.76
HDL: ¯x±s , mmol/L	1.27±0.37
LDL:¯x±s , mmol/L	2.51±0.77

TC, total cholesterol; TG, triglyceride; HDL, high density lipoprotein; LDL, low density lipoprotein.

### 3.2 Comparison of TC, TG, HDL-C, LDL-C, TC/TG, LDL-C/HDL-C between choledocholithiasis patients group and control group

There were 64 males and 105 females in choledocholithiasis patients group, while 74 males and 95 females in control group. The difference wasn’t statistically significant (*P*=0.268). Similarly, there was no statistically significant difference in age between the two groups (*P*=0.927). There were significant differences in TC (*P*<0.001), TG (*P*=0.006), HDL-C (*P*<0.001) and LDL-C (*P*=0.001) between the two groups. But there were no significant differences in TC/TG (*P*=0.516) and LDL-C/HDL-C (*P*=0.139). The TC, TG, HDL-C, LDL-C in the choledocholithiasis patients group were significantly lower than that in the healthy population. (**Table 2**).

**Table 2:** Analysis of the differences between choledocholithiasis group and control group.

Parameters	choledocholithiasis group n=169	control group n=169	*P*
Sex (M/F)	64/105	74/95	0.268
Age (years): ¯x±s	56.26±10.80	56.15±10.53	0.927
TC:¯x±s , mmol/L	4.30±1.03	4.96±0.97	<0.001
TG: ¯x±s , mmol/L	1.33±0.76	1.61±1.10	0.006
HDL: ¯x±s , mmol/L	1.27±0.37	1.44±0.34	<0.001
LDL:¯x±s , mmol/L	2.51±0.77	2.79±0.75	0.001
TC/TG:¯x±s	4.20±2.53	4.03±2.07	0.516
LDL/HDL: ¯x±s	2.16±0.95	2.02±0.69	0.139

TC, total cholesterol; TG, triglyceride; HDL, high density lipoprotein; LDL, low density lipoprotein.

### 3.3 Whether there were differences between the choledocholithiasis patients group and the control group by gender

In the subgroup analysis, we divided both groups again into male and female groups to compare the above parameters for differences. There was no significant difference in age (*P*=0.313), TG (*P*=0.182), TC/TG (*P*=0.982), LDL-C/HDL-C (*P*=0.392) between the male choledocholithiasis patients group and the male healthy population group. There were significant differences in TC (*P*=0.001), HDL-C (*P*=0.014) and LDL-C (*P*=0.026) between the two groups. There was no significant difference in age (*P*=0.317), TC/TG (*P*=0.590), LDL-C/HDL-C (*P*=0.116) between the female choledocholithiasis patients group and the female healthy population group. There were significant differences in TC (*P*<0.001), TG (*P*=0.016), HDL-C (*P*<0.001) and LDL-C (*P*=0.009) between the two groups. The results of male choledocholithiasis patients were consistent with those of female choledocholithiasis patients, except for difference in TG. (**Table 3**).

**Table 3:** Whether there were differences between the choledocholithiasis group and the control group by gender.

Parameters	Sex:male			Sex:female		
	choledocholithiasis group n=64	control group n=74	*P*	choledocholithiasis group n=105	control group n=95	*P*
Age (years): ¯x±s	55.09±11.33	57.00±10.77	0.313	56.97±10.44	55.49±10.34	0.317
TC:¯x±s , mmol/L	4.13±1.16	4.75±1.02	0.001	4.40±0.93	5.13±0.90	<0.001
TG: ¯x±s , mmol/L	1.44±0.91	1.69±1.20	0.182	1.26±0.65	1.56±1.01	0.016
HDL: ¯x±s , mmol/L	1.16±0.37	1.30±0.30	0.014	1.33±0.35	1.55±0.33	<0.001
LDL:¯x±s , mmol/L	2.42±0.84	2.73±0.80	0.026	2.56±0.72	2.83±0.72	0.009
TC/TG:¯x±s	3.61±1.77	3.61±1.76	0.982	4.55±2.85	4.36±2.24	0.590
LDL/HDL: ¯x±s	2.34±1.15	2.20±0.78	0.392	2.04±0.80	1.89±0.58	0.116

TC, total cholesterol; TG, triglyceride; HDL, high density lipoprotein; LDL, low density lipoprotein.

## 4 Discussion

Since Poulletier Delasalle first isolated cholesterol from human gallstones in 1769, its unique physiological and pathological effects have been extensively studied (16). Cholesterol has three basic biological functions: cell signaling, hormone synthesis and metabolic homeostasis (17). Cholesterol is an important and indispensable substance in animal tissue cells, it not only participates in the formation of cell membranes, but also is the raw material for the synthesis of bile acids, vitamin D and steroid hormones. Cholesterol can also be metabolized into bile acids, steroid hormones, 7-dehydrocholesterol, and 7-dehydrocholesterol is converted into vitamin D3 when exposed to ultraviolet light. On the other hand, excess cholesterol can be harmful or even fatal to nucleated cells (18). Because the accumulation of cholesterol promotes the activation of innate and adaptive inflammatory responses (19). Previous studies had established high plasma cholesterol levels as a causal risk factor for atherosclerosis and cardiovascular disease (20). A study indicated that higher serum levels of total cholesterol and LDL-C were associated with an increased severity of acute pancreatitis (21). A study showed that abnormal liver cholesterol metabolism, such as excessive liver cholesterol biosynthesis, liver cholesterol intake or output interference, liver cholesterol esterification abnormalities, can cause cholesterol gallstones (22). Since abnormal cholesterol metabolism is associated with a variety of diseases, and the main component of secondary choledocholithiasis is also cholesterol, is choledocholithiasis also caused by abnormal cholesterol metabolism?

This study found that serum total cholesterol, HDL-C and LDL-C levels in both male and female choledocholithiasis patients were significantly lower than those in healthy people. We suspect that the cause of this result may be abnormal liver cholesterol metabolism. Previous studies have confirmed that cholesterol supersaturation in bile is related to the increase of liver and plasma cholesterol content, which is the main pathogenic factor of gallstone (23, 24). Since various Niemann-Pick C1-like 1 (NPC1L1) mutations have been found to increase biliary cholesterol concentrations, which in turn increases the risk of gallstone, the abundance of NPC1L1 proteins in the human liver is considered a plausible defense against gallstone (25). In other words, the human liver has NPC1L1, which reduces the excretion of biliary cholesterol. Therefore, when the expression of NPC1L1 in the liver is reduced, it may lead to the supersaturation of cholesterol in the secreted bile, which makes the formation of choledocholithiasis more likely. Because more cholesterol is discharged through bile, the plasma cholesterol is relatively reduced, which is why the serum total cholesterol, HDL-C and LDL-C of choledocholithiasis patients are significantly lower than that of healthy people.

In this study, although the triglyceride level of the male choledocholithiasis patients group was lower than that of the healthy population group, but there was no statistically significant difference. The reason for this result may be that there were only 64 male choledocholithiasis patients in this study, resulting in no significant difference in results. However, 105 female choledocholithiasis patients were included in this study, nearly twice as the male choledocholithiasis patients, so the triglyceride level of female choledocholithiasis patients was significantly different from that of the normal control group. Therefore, subsequent studies involving more male patients with choledocholithiasis are needed to prove whether triglyceride levels in male patients with choledocholithiasis are significantly lower than those in healthy people.

In summary, the present study confirmed that cholesterol level (including TC, HDL-C and LDL-C) in patients with choledocholithiasis was significantly lower than those in healthy people, and this difference was independent of sex. A study (26) published in 2024 showed that TC, HDL-C and LDL-C were negatively correlated with cholelithiasis, and HDL-C and LDL-C were negatively correlated linearly. The study also found that lower TC and higher TG levels were two independent causal risk factors. This study confirmed that the cholesterol level of choledocholithiasis patients was significantly lower than that of healthy people, which was basically consistent with the above study. However, neither this study nor the above-mentioned study has delved deeply into the nature of choledocholithiasis and whether the cholesterol content in bile is higher than that in healthy people, thereby proving that the serum cholesterol content in patients with choledocholithiasis is lower than that in healthy people because more cholesterol enters bile. And there is no research on this at present. We look forward to having research on this in the future.

This study has some limitations. First, this study only found that the serum cholesterol level of choledocholithiasis patients was significantly lower than that of healthy people, and did not study whether the bile cholesterol content of choledocholithiasis patients was significantly higher than that of healthy people. Secondly, patients with cardiovascular and cerebrovascular diseases and taking lipid-lowering drugs were excluded, and these people were basically over 70 years old, so people over 70 years old were not included. In addition, the study excluded patients who had prior cholecystectomy, which may have influenced the results. And there is no clear standard to distinguish whether choledocholithiasis are primary or secondary. Therefore, some of the patients with choledocholithiasis included in this study were complicated with gallbladder stones, which might have an impact on the results. Last, this study was a single-center retrospective study with a small sample size. Therefore, it is hoped that further studies will be conducted in the future and more patients will be included. Whether abnormal cholesterol metabolism is related to the formation of choledocholithiasis and its mechanism, whether taking lipid-lowering drugs can reduce the incidence of choledocholithiasis remains to be studied.

In conclusion, the results of this study are of great clinical significance. The serum TC, HDL-C and LDL-C in choledocholithiasis patients were significantly lower than those in healthy people, and TG in female patients was significantly lower than that in healthy people, but there was no difference in male patients.

## Data Availability

The raw data supporting the conclusions of this article will be made available by the authors, without undue reservation.

## References

[1] Copelan A Kapoor BS. Choledocholithiasis: diagnosis and management. Tech Vasc Interv Radiol. (2015) 18:244–55. doi: 10.1053/j.tvir.2015.07.008 26615165

[2] Möller M Gustafsson U Rasmussen F Persson G Thorell A. Natural course vs interventions to clear common bile duct stones: data from the Swedish Registry for Gallstone Surgery and Endoscopic Retrograde Cholangiopancreatography (GallRiks). JAMA Surg. (2014) 149:1008–13. doi: 10.1001/jamasurg.2014.249 25133326

[3] Collins C Maguire D Ireland A Fitzgerald E Sullivan GC. A prospective study of common bile duct calculi in patients undergoing laparoscopic cholecystectomy: natural history of choledocholithiasis revisited. Ann Surg. (2004) 239:28–33. doi: 10.1097/01.sla.0000103069.00170.9c 14685097 PMC1356189

[4] Murison MS Gartell PC McGinn FP. Does selective peroperative cholangiography result in missed common bile duct stones. J R Coll Surg Edinb. (1993) 38:220–4. 7693932

[5] Ko CW Lee SP. Epidemiology and natural history of common bile duct stones and prediction of disease. Gastrointest Endosc. (2002) 56:S165–9. doi: 10.1016/S0016-5107(02)70005-9 12447261

[6] Wilkins T Agabin E Varghese J Talukder A. Gallbladder dysfunction: cholecystitis, choledocholithiasis, cholangitis, and biliary dyskinesia. Prim Care. (2017) 44:575–97. doi: 10.1016/j.pop.2017.07.002 29132521

[7] Williams EJ Green J Beckingham I Parks R Martin D Lombard M. Guidelines on the management of common bile duct stones (CBDS). Gut. (2008) 57:1004–21. doi: 10.1136/gut.2007.121657 18321943

[8] Park SM Kim WS Bae IH Kim JH Ryu DH Jang LC. Common bile duct dilatation after cholecystectomy: a one-year prospective study. J Korean Surg Soc. (2012) 83:97–101. doi: 10.4174/jkss.2012.83.2.97 22880184 PMC3412191

[9] Maple JT Ben-Menachem T Anderson MA Appalaneni V Banerjee S Cash BD. The role of endoscopy in the evaluation of suspected choledocholithiasis. Gastrointest Endosc. (2010) 71:1–9. doi: 10.1016/j.gie.2009.09.041 20105473

[10] Tseng CW Chen CC Chen TS Chang FY Lin HC Lee SD. Can computed tomography with coronal reconstruction improve the diagnosis of choledocholithiasis. J Gastroenterol Hepatol. (2008) 23:1586–9. doi: 10.1111/j.1440-1746.2008.05547.x 18713297

[11] Tang G Zhang J Chen R Zhang J Zhou R. Percutaneous transhepatic cholangioscopy combined with endoscopic retrograde cholangiopancreatography for bilateral biliary bridge drainage for Malignant biliary obstruction. Endoscopy. (2024) 56:E724–5. doi: 10.1055/a-2375-0187 PMC11309847 39117329

[12] Martín Arnau AB Molera Espelt A Villaba Auñon J Sánchez-Cabús S. Percutaneous transhepatic cholangioscopy in the management of hepatolithiasis. Cir Esp (Engl Ed). (2024) 102(11):599–604. doi: 10.1016/j.cireng.2024.06.011 39089368

[13] Giljaca V Gurusamy KS Takwoingi Y Higgie D Poropat G Stimac D. Endoscopic ultrasound versus magnetic resonance cholangiopancreatography for common bile duct stones. Cochrane Database Syst Rev. (2015) 2015:CD011549. doi: 10.1002/14651858.CD011549 25719224 PMC6464848

[14] Cortes VA Busso D Maiz A Arteaga A Nervi F Rigotti A. Physiological and pathological implications of cholesterol. Front Biosci (Landmark Ed). (2014) 19:416–28. doi: 10.2741/4216 24389193

[15] Weissberg PL Bennett MR. Atherosclerosis–an inflammatory disease. N Engl J Med. (1999) 340:1928–9. doi: 10.1056/NEJM199901143400207 10375320

[16] Luo J Yang H Song BL. Mechanisms and regulation of cholesterol homeostasis. Nat Rev Mol Cell Biol. (2020) 21:225–45. doi: 10.1038/s41580-019-0190-7 31848472

[17] Lingwood D Simons K. Lipid rafts as a membrane-organizing principle. Science. (2010) 327:46–50. doi: 10.1126/science.1174621 20044567

[18] Yvan-Charvet L Bonacina F Guinamard RR Norata GD. Immunometabolic function of cholesterol in cardiovascular disease and beyond. Cardiovasc Res. (2019) 115:1393–407. doi: 10.1093/cvr/cvz127 31095280

[19] Tall AR Yvan-Charvet L. Cholesterol, inflammation and innate immunity. Nat Rev Immunol. (2015) 15:104–16. doi: 10.1038/nri3793 PMC4669071 25614320

[20] Ference BA Kastelein J Ginsberg HN Chapman MJ Nicholls SJ Ray KK. Association of genetic variants related to CETP inhibitors and statins with lipoprotein levels and cardiovascular risk. JAMA. (2017) 318:947–56. doi: 10.1001/jama.2017.11467 PMC5710502 28846118

[21] Zhou X Jin S Pan J Lin Q Yang S Lu Y. Relationship between cholesterol-related lipids and severe acute pancreatitis: from bench to bedside. J Clin Med. (2023) 12:1729. doi: 10.3390/jcm12051729 36902516 PMC10003000

[22] Zhang C Dai W Yang S Wu S Kong J. Resistance to cholesterol gallstone disease: hepatic cholesterol metabolism. J Clin Endocrinol Metab. (2024) 109:912–23. doi: 10.1210/clinem/dgad528 37668355

[23] Schäfer C Schuh A Rau BM. Diagnosis and therapy of gallstone disease. MMW Fortschr Med. (2021) 163:40–5. doi: 10.1007/s15006-020-9525-8 33783796

[24] Wang DQ Carey MC. Complete mapping of crystallization pathways during cholesterol precipitation from model bile: influence of physical-chemical variables of pathophysiologic relevance and identification of a stable liquid crystalline state in cold, dilute and hydrophilic bile salt-containing systems. J Lipid Res. (1996) 37:606–30. doi: 10.1016/S0022-2275(20)37603-3 8728323

[25] Krawczyk M Niewiadomska O Jankowska I Jankowski K Więckowski S Lebensztejn D. Common variant p.D19H of the hepatobiliary sterol transporter ABCG8 increases the risk of gallstones in children. Liver Int. (2022) 42:1585–92. doi: 10.1111/liv.15186 35129276

[26] Chen L Qiu W Sun X Gao M Zhao Y Li M. Novel insights into causal effects of serum lipids and lipid-modifying targets on cholelithiasis. Gut. (2024) 73:521–32. doi: 10.1136/gutjnl-2023-330784 37945330

